# How to Minimize Hyper-Continence After Intracorporeal Robotic Neobladder Configuration in Women? The Three-Layer Posterior Reconstruction During Florence Robotic IntraCorporeal Neobladder (FloRIN)

**DOI:** 10.3390/jcm14238397

**Published:** 2025-11-26

**Authors:** Fabrizio Di Maida, Luca Lambertini, Antonio Andrea Grosso, Vincenzo Salamone, Daniele Paganelli, Laura Di Stefano, Francesca Conte, Filippo Lipparini, Matteo Salvi, Rino Oriti, Andrea Mari, Andrea Minervini

**Affiliations:** 1 Department of Experimental and Clinical Medicine, University of Florence, 50134 Florence, Italy; luca.lambertini@unifi.it (L.L.); antonioandrea.grosso@unifi.it (A.A.G.); vincenzo.salamone@unifi.it (V.S.); daniele.paganelli@unifi.it (D.P.); laura.distefano@unifi.it (L.D.S.); francesca.conte1@unifi.it (F.C.); filippo.lipparini@unifi.it (F.L.); matteo.salvi@unifi.it (M.S.); rino.oriti@unifi.it (R.O.); andrea.mari@unifi.it (A.M.); andrea.minervini@unifi.it (A.M.); 2 Unit of Oncologic Minimally-Invasive Urology and Andrology, Careggi Hospital, 50134 Florence, Italy

**Keywords:** continence, FloRIN, neobladder, posterior reconstruction, robotic

## Abstract

**Background:** Orthotopic neobladder in women presents functional challenges, notably hyper-continence requiring intermittent self-catheterization. Here, we describe a three-layer urethro-ileal anastomosis incorporating a peritoneal flap and evaluate its impact on functional outcomes at the time of robot-assisted radical cystectomy (RARC) and Florence Robotic Intracorporeal Neobladder (FloRIN) reconfiguration. **Methods:** Clinical data from consecutive patients treated with non-sexual-sparing RARC and FloRIN were prospectively collected between March 2016 and January 2024. Hyper-continence was defined as intermittent self-catheterization or postvoid residual > 200 cc at last follow-up. A three-layer anastomosis was performed incorporating a peritoneal flap and utilizing a three-step barbed suture technique for posterior reconstruction: (1) the peritoneum of the rectouterine pouch was sutured to the paraurethral ligaments, (2) the external ileal layer was approximated to the paraurethral ligaments, and (3) the ileal mucosa was anastomosed to the urethra. Urodynamic evaluation was performed 6 months postoperatively. **Results:** Overall, 32 patients entered the study. Median age was 72 (IQR 67–75) years, and median BMI was 24 (IQR 22–27) kg/m^2^. Preoperative incontinence was reported in 10% of cases, while no hyper-continence was recorded preoperatively. Median console time was 334 (IQR 305–363) minutes. Early major complications (<30 days) occurred in three (9.4%) patients, while delayed major complications (>30 days) were observed in four (12.5%) cases. After a median follow-up of 36 (IQR 30–42) months, hyper-continence was observed in 7.2% of patients with a median self-catheterization number of 3 (IQR 2–5) per day. Multivariable analysis confirmed BMI > 25 kg/m^2^ (OR: 1.24, *p* = 0.03) and age > 70 years (OR: 1.18, *p* = 0.04) as independent predictors of hyper-continence after FloRIN. **Conclusions:** Robot-assisted three-layer posterior reconstruction during FloRIN configuration in female patients demonstrated low rates of hyper-continence and no cases of permanent catheterization in long-term follow-up.

## 1. Introduction

The gold standard treatment for patients with high-risk BCG-unresponsive non-muscle-invasive bladder cancer and muscle-invasive bladder cancer is radical cystectomy (RC) with urinary diversion [1]. Orthotopic neobladder (ONB) is considered the optimal form of urinary diversion, as it most closely mimics the function of the native bladder. Specifically, robot-assisted radical cystectomy (RARC) with intracorporeal ONB formation represents an innovative approach to bladder cancer treatment for both men and women [2]. In recent years, this technique has become increasingly common and is now regarded as the standard of care in many institutions [3,4,5,6,7]. However, in female patients, managing postoperative urinary continence remains a significant challenge [8]. Women experience long-term complications distinct from those observed in men, particularly in terms of voiding dysfunction and chronic urinary retention, with reported rates ranging from 31% to 61% in various studies [7,9].

Hyper-continence in women undergoing ONB reconstruction is a well-recognized functional complication, typically defined as the inability to void or voiding with a significant postvoid residual volume, generally exceeding 150–200 mL. This condition reflects impaired neobladder emptying and often necessitates intermittent self-catheterization. Importantly, women appear to be disproportionately affected compared with men, with reported incidences approaching 50% in contemporary series and systematic reviews [10]. Female pelvic anatomy and innervation contribute to these complications [11,12], with the primary mechanism leading to hyper-continence and chronic urinary retention appearing to be a deficiency in posterior support of the neobladder. This deficiency results in posterior migration of the neobladder and kinking of the neobladder–urethral anastomosis. Over the years, various posterior reconstruction techniques have been introduced and directly translated into the robotic scenario [13,14,15]. However, although hyper-continence and chronic urinary retention are common postoperative complications following intracorporeal ONB reconstruction in women, predictive factors and standardized surgical techniques to prevent or mitigate these issues remain lacking.

To address this gap, the present study aimed to analyze surgical and urodynamic data from consecutive female patients treated with non-sexual-sparing RARC and intracorporeal ONB reconstruction using the Florence Robotic Intracorporeal Neobladder (FloRIN) technique [16,17]. In particular, we focused on evaluating how a three-layer posterior reconstruction of the ileo-urethral anastomosis may enhance posterior support and reduce the incidence of hyper-continence and chronic urinary retention. Additionally, we aimed to evaluate whether any clinical or surgical predictors of postoperative hyper-continence exist, with the goal of improving patient selection and preoperative counseling.

## 2. Materials and Methods

### 2.1. Patient and Dataset

Clinical and surgical data of all consecutive patients treated from March 2016 to January 2024 with RARC, lymph node dissection (LND), and FloRIN reconfiguration at a single tertiary referral center were prospectively collected. For the present study, we only evaluated RARC in female patients with non-organ-sparing surgery and with a three-layer posterior reconstruction with the interposition of a peritoneal flap. In our series, non-organ-sparing surgery refers to the removal of the reproductive organs (uterus, fallopian tubes, ovaries, and anterior vaginal wall) during RC, which is the standard approach in women without specific indications for organ preservation. Patient demographics and preoperative features were collected, including age, BMI, ASA score, age-adjusted Charlson Comorbidity Index (CCI), neo-adjuvant chemotherapy, and previous abdominal surgery. Preoperative urinary incontinence and presence of pelvic organ prolapse (POP) according to the Halfway system classification (HWS) were recorded. The preoperative evaluation included contrast-enhanced computed tomography (CT) of the chest and abdomen. Peri- and postoperative outcomes, including operative time, conversion rate, intraoperative complication rate, and blood transfusions, estimated blood loss, time to canalization, and alterations in creatinine and hemoglobin in the first postoperative days, were thoroughly collected. The follow-up schedule included blood analysis and CT scans performed three months after surgery, then every 6 months. The functional evaluation was based on the determination of the postvoid residual (PVR) with extemporaneous catheterization and on the urodynamic assessment performed 6 months after surgery. Hyper-continence status was defined as the presence of at least two intermittent self-catheterizations per day or PVR > 200 cc at last follow-up assessment. The transurethral catheter was routinely removed on postoperative day 21 without any prior imaging. Cystography was requested only when, based on intraoperative assessment, the operating surgeon judged that the descent of the ileal segment was difficult or that the urethro-ileal anastomosis was not entirely tension-free.

### 2.2. Surgical Technique

**Figure 1:**
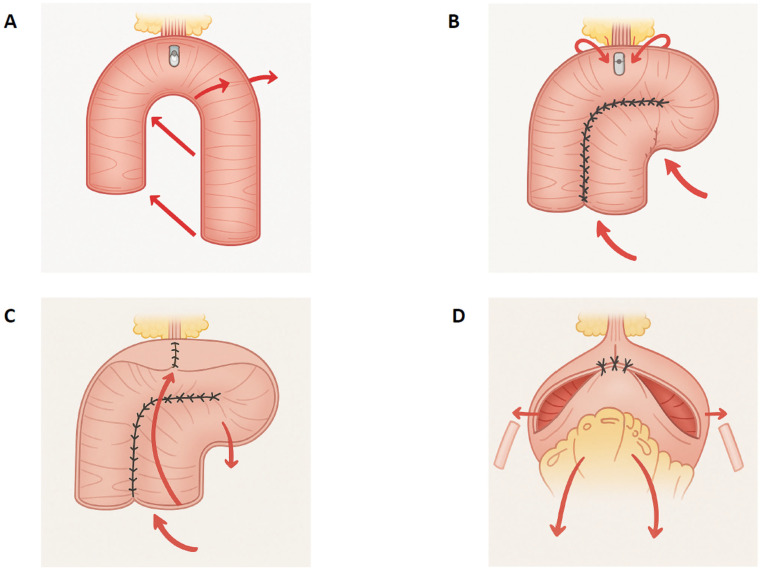
Surgical stages of FloRIN reconfiguration (**A**) Perform the urethro-ileal anastomosis to create an asymmetrical “U” shape, with approximately 25–30 cm distal and 20 cm proximal to the anastomosis. (**B**) Reshape the posterior plate into an “L” configuration by suturing the arms of the “U” in parallel alignment, positioning the distal loop to the right to form the short arm of the “L.” (**C**) Reconstruct the neobladder neck by suturing a 2–5 cm longitudinal segment upward from the 12 o’clock point of the anastomosis, then fold the posterior plate anteriorly. (**D**) Reimplant both ureters in an orthotopic position and close the anterior plate using an inverted “V”-shaped suture.

The Da Vinci Si or X robotic platform (Intuitive Surgical, Sunnyvale, CA, USA) was used for all procedures, employing a four-arm setup with a 0° laparoscope. The FloRIN surgical technique has been detailed previously [16] [Figure 1].

**Figure 2:**
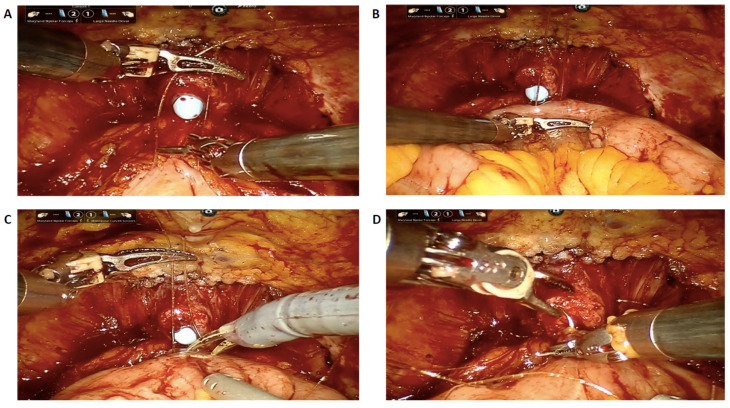
Surgical steps of the three-layer posterior reconstruction during Florence Robotic IntraCorporeal Neobladder (FloRIN). (**A**) Suture between the posterior Douglas peritoneum and the posterior paraurethral ligaments; (**B**) Suture between the posterior external ileal layer and the posterior paraurethral ligaments; (**C**) Ileal incision along the antimesenteric border for subsequent urethro-ileal anastomosis; (**D**) Suture between the ileal mucosa and the urethra and completion of the anastomosis.

Briefly, the patient is positioned in a 30° Trendelenburg using a standard six-port transperitoneal layout, which is then adjusted to 20° after completion of the extirpative phase to optimize bowel manipulation and the urethro-neobladder anastomosis. The principal steps of the reconstructive phase are as follows. (1) Isolation of 50 cm of the ileum. (2) Performing the urethro-ileal anastomosis to form an asymmetrical U-configuration (25 cm distal and 20 cm proximal to the anastomosis). In female patients, to avoid the state of hyper-continence, a three-layer posterior reconstruction is performed with the interposition of a peritoneal flap and the three-layer anastomosis posterior is carried out with a three-step barbed suture: (A) Peritoneum of the rectouterine pouch–Posterior paraurethral ligaments; (B) Posterior external ileal layer–Posterior paraurethral ligaments; (C) Ileal mucosae–Urethra [Figure 2]. (3) The ileal segment is transected using an Endo-GIA 60-mm Echelon Powered Endopath Stapler (Ethicon Inc., Cincinnati, OH, USA). Intestinal continuity is subsequently re-established via an intracorporeal side-to-side anastomosis created with a single longitudinal firing. The two transverse enterotomies are then closed using a double-layer running 3-0 Stratafix suture. (4) Detubularization of the asymmetrical U-shaped ileal segment is performed. The posterior plate is then reshaped into an “L” configuration by suturing the parallel limbs of the U together and positioning the extended loop segment distally to the right, forming the short limb of the “L”. (5) The neobladder neck is fashioned by suturing a 2–5 cm tract longitudinally from the 12 o’clock position of the urethro-ileal anastomosis upward. (6) The posterior plate is then folded anteriorly—from distal to proximal—approximately 5 cm to the right of the proximal margin of the posterior closure to generate two symmetrical components. (7) The ureters are reimplanted bilaterally in an orthotopic, direct fashion without anti-reflux mechanisms, each positioned on the lateral aspect of the corresponding anterior segment. (8) The anterior plate is closed using an inverted V-shaped suture line.

### 2.3. Urodynamic Study

Continence outcomes were assessed six months after the FloRIN reconstruction. All patients received comprehensive instruction on pelvic floor training following transurethral catheter removal and were advised to empty the neobladder every 2–3 h, both during the day and at night. Daytime and nighttime continence were defined as the use of ≤1 pad per respective period. Beginning with the initial case, patients were offered a pressure flow study (PFS) at six months postoperatively to assess neobladder functional performance. Non-invasive uroflowmetry (NIF) and postvoid residual (PVR) measurements were obtained prior to initiating PFS. The PFS was conducted in accordance with our standardized urodynamic protocol for patients undergoing RARC with FloRIN neobladder reconstruction [18,19]. A water-filled 6 Fr dual-lumen urethral catheter and a 12 Fr water-charged rectal catheter with an introducer were utilized. Patients were positioned in a semi-seated posture, and the neobladder was infused with room-temperature normal saline at a rate of 30 mL/min. Maximal bladder capacity was defined as the point at which the patient first experienced abdominal discomfort or demonstrated urinary leakage. Compliance was calculated as the change in intraluminal volume divided by the corresponding rise in neobladder pressure (mL/cmH_2_O) during the filling phase. Although no universal standard exists for ideal neobladder compliance, proposed thresholds suggest that values exceeding 12.5–30 mL/cmH_2_O may represent the lower boundary of normal [20,21]. If a sudden rise or fluctuation in neobladder pressure occurred, measurements were taken only after the pressure tracing had stabilized. Provocative maneuvers (Valsalva and cough) were performed to evaluate stress urinary incontinence and to determine the abdominal leak-point pressure (ALPP). Patients were then instructed to void in a comfortable position. During the voiding phase of the PFS, the maximum flow rate was recorded, followed by assessment of the PVR. After completion of voiding, a urethral pressure profile was obtained using a perfusion rate of 5 mL/min and a catheter withdrawal speed of 2 mm/s to accurately determine the maximal urethral closure pressure (MUCP).

### 2.4. Statistical Analysis

For analytic purposes, all available patient- and tumor-related variables from our institutional database were considered as independent predictors. Initially, descriptive statistics were generated, reporting medians with interquartile ranges for continuous variables and frequencies with corresponding proportions for categorical variables. Continuous variables were compared using either the Student *t*-test or the Mann–Whitney U test, depending on whether their distribution met normality assumptions (assessed via the Kolmogorov–Smirnov test). Categorical variables were evaluated using the Chi-square test. A multivariable logistic regression model was then constructed to identify clinical predictors of postoperative hyper-continence. Statistical analyses were conducted using SPSS version 29 (IBM SPSS Statistics for Mac, Armonk, NY, USA, IBM Corp). All tests were two-sided, with statistical significance defined as *p* < 0.05.

## 3. Results

A total of 189 patients underwent RARC with FloRIN reconfiguration, of whom 32 were women with non-organ-sparing surgery and were included in the final analysis. Preoperative characteristics are summarized in Table 1. The median age was 72 (IQR 67–75) years, and the median BMI was 24 (IQR 22–27) kg/m^2^. The majority of patients (62.6%) were former smokers, while 21.8% were never smokers and 15.6% were current smokers. Neoadjuvant chemotherapy was administered to 23 (71.9%) patients, and the median age-adjusted CCI was 3 (IQR 3–4). Regarding preoperative clinical staging, 28 (87.5%) patients had clinically localized disease (cT ≤ 2). Three (9.4%) patients had radiologically detectable lymph node involvement (cN1) on preoperative imaging. Preoperative POP > III was observed in 2 (6.3%) patients, while baseline urinary incontinence was reported in 3 (9.3%) patients. No patient exhibited preoperative urinary retention, elevated PVR, or clinical evidence of being retention-prone. This excluded the possibility that pre-existing functional or anatomical abnormalities could have confounded postoperative hyper-continence outcomes.

Intraoperative and perioperative data are detailed in Table 2. The median console time was 331 (IQR 308–347) minutes. No major intraoperative complications occurred, and no conversions to open surgery were required. The median estimated blood loss was 330 (IQR 260–390) mL, and no intraoperative blood transfusions were needed. Postoperative recovery was uneventful in most cases, with a median time to canalization of 5 (IQR 4–7) days. The median hemoglobin decrease from baseline was 2.7 (IQR 1.8–3.5) g/dL, while the median change in creatinine from baseline to the third postoperative day was 0.5 (IQR 0.2–1.2) mg/dL. Notably, five (15.6%) patients completed the procedure without ureteral stent placement. Details of major postoperative complications (Clavien–Dindo grade ≥ 3) are presented in Table 2. Early major complications (<30 days) occurred in 3 (9.4%) patients, while delayed major complications (>30 days) were observed in 4 (12.5%) cases. Readmission within 30 days occurred in 2 (6.3%) patients. Renal function was stable over time, with a median creatinine change of 0.12 (IQR 0.03–0.41) mg/dL between discharge and 3-month follow-up, and 0.11 (IQR 0.04–0.43) mg/dL at 6-month assessment. Hydronephrosis was observed in 2 (6.3%) patients at 3-month follow-up CT imaging.

Functional outcomes are reported in Table 3. The median follow-up was 36 (IQR 30–42) months. No cases of permanent catheterization were reported. At urodynamic evaluation, the median first desire to void was 190 mL (IQR 170–240), while median compliance reached 22 mL/cmH_2_O (IQR 18–25), indicating adequate neobladder elasticity. The median maximum flow rate (Qmax) was 17 mL/s (IQR 14–25), and the median average flow rate (Qave) was 11 mL/s (IQR 7–18). PVR was low with a median value of 40 mL (IQR 30–55). Abdominal leak point pressure (ALPP) was observed in 28.1% of patients, with a median minimum volume of 175 mL (IQR 145–230) and a median minimum pressure of 47 cmH_2_O (IQR 38–55). Phasic neobladder contractions were present in 25% of cases. Urethral pressure profile revealed a median MUCP of 44 (OQR 32–58) cmH_2_O. Hyper-continence occurred in 9.4% of patients, with a median self-catheterization frequency of 3 times/day (IQR 2–5).

Multivariate analysis [Table 4] identified BMI > 25 kg/m^2^ and age > 70 years as independent predictors of postoperative hyper-continence. Specifically, patients with a BMI > 25 kg/m^2^ had a 24% higher risk of developing hyper-continence compared to those with lower BMI values (OR 1.24, 95% CI: 1.12–1.39, *p* = 0.03). Similarly, patients aged over 70 years showed an 18% increased risk compared to younger counterparts (OR 1.18, 95% CI: 1.09–1.73, *p* = 0.04). ASA score ≥ 3 was associated with hyper-continence in univariate analysis (OR 1.12, 95% CI: 1.15–1.92, *p* = 0.04) but lost statistical significance in the multivariate model (OR 1.02, 95% CI: 0.98–1.11, *p* = 0.23)

## 4. Discussion

RARC with intracorporeal neobladder reconstruction has gained increasing acceptance as a viable alternative to open surgery, particularly in female patients, where the benefits of minimally invasive approaches are increasingly recognized [22,23]. However, functional outcomes, particularly in terms of voiding efficiency and hyper-continence, remain a major concern in this patient population [10,24]. Women undergoing non-organ-sparing radical cystectomy lack the uterine, vaginal, and parametrial support that normally stabilizes the bladder neck and pelvic axis. This loss may predispose to impaired neobladder emptying and hyper-continence. By contrast, organ-sparing approaches preserve these structures and may favor better voiding function [25]. The underlying mechanism of hyper-continence in women is considered multifactorial. The absence of posterior support may facilitate posterior migration of the neobladder and kinking of the urethro-ileal anastomosis. Unlike men, who benefit from the structural support of Denonvilliers’ fascia, women lack a comparable posterior anchoring plane [26]. Several technical modifications have been proposed to enhance support in female ONB reconstruction, including round ligament suspension and alternative detubularization strategies. However, results remain inconsistent, and some structures—such as the round ligament—may lose elasticity over time, limiting their long-term stabilizing value [27].

Our study focuses on a structured three-layer posterior reconstruction that incorporates a peritoneal flap and provides immediate anchoring between the rectouterine pouch, paraurethral ligaments, and the external ileal layer. The present series shows encouraging outcomes: no cases of permanent catheterization and a 9.4% rate of hyper-continence after a median follow-up of 36 months. Even among patients requiring intermittent catheterization, the median frequency remained low (3/day), suggesting partial preservation of spontaneous voiding. Urodynamic assessments further corroborated these findings, demonstrating satisfactory voiding parameters. The median maximum flow rate (Qmax) of 17 mL/s and compliance of 22 mL/cmH_2_O are indicative of a well-functioning reservoir with sufficient compliance and emptying efficiency. The median PVR of 40 mL is within an acceptable range and suggests that the majority of patients were able to void efficiently without significant retention. These findings highlight the potential advantages of structured posterior support in preventing neobladder outlet obstruction and optimizing detrusor function. Direct comparison of hyper-continence rates across different intracorporeal neobladder configurations is inherently difficult, as the available literature does not stratify functional outcomes by sex. In the analysis by Piramide et al. [3], the need for intermittent self-catheterization ranges from 3% to 11% across the major intracorporeal techniques; however, these data derive from mixed cohorts predominantly composed of male patients and therefore cannot be reliably extrapolated to women.

We also evaluated whether any clinical or surgical predictors of postoperative hyper-continence could be identified. Multivariable analysis revealed two independent predictors: BMI > 25 kg/m^2^ and age > 70 years. A higher BMI has previously been linked to pelvic floor dysfunction, as increased intra-abdominal pressure can alter voiding mechanics and impair neobladder function; moreover, obesity may promote fibrosis and reduce neobladder compliance, further contributing to voiding difficulties [25]. Age-related changes in pelvic support and reduced reservoir contractility likely explain the association between age >70 years and hyper-continence, as elderly patients may generate lower voiding pressures and are more vulnerable to the lack of posterior support after female neobladder reconstruction [28]. These findings suggest that patient selection and preoperative counseling should emphasize these risk factors, and older patients with higher BMI may benefit from closer postoperative monitoring and early interventions to prevent chronic retention.

There are several limitations to this study. The sample size is relatively small, limiting the generalizability of our findings. Additionally, the lack of a comparative cohort prevents definitive conclusions regarding the superiority of this approach over other reconstruction techniques. A further point concerns the long-term durability of the three-layer posterior reconstruction. Although the structural anchoring between the peritoneum of the rectouterine pouch and the external ileal layer theoretically provides stable support, hyper-continence in women tends to appear early, suggesting that the critical determinant is the initial posterior stabilization of the urethro-ileal anastomosis rather than late fibrotic remodeling. Nonetheless, long-term studies are needed to determine whether this reconstructive step maintains its mechanical effectiveness over time. Finally, the lack of standardized urodynamic criteria for ONBs complicates postoperative functional interpretation. In particular, the physiological value of urethral pressure profiles in neobladder patients may be limited by the absence of the native bladder neck, altered urethral support, and variable pressure transmission of the ileal reservoir.

Acknowledging these limitations, the findings from this study have important clinical implications. First, they provide further support for the safety and feasibility of robotic intracorporeal neobladder reconstruction in women, demonstrating favorable perioperative and long-term functional outcomes. Second, they underscore the importance of structured posterior support in mitigating hyper-continence, which remains a major functional limitation in female patients undergoing orthotopic diversion. Future research should focus on larger, multi-institutional studies to validate the benefits of this approach in a broader population. Additionally, prospective comparative trials evaluating different reconstruction techniques (e.g., round ligament suspension vs. three-layer posterior reconstruction) would be valuable in identifying the optimal surgical strategy. Longitudinal follow-up with detailed urodynamic assessments is also warranted to assess the durability of functional outcomes over time.

## 5. Conclusions

This study demonstrates that robot-assisted three-layer posterior reconstruction during FloRIN configuration in female patients is a feasible and effective approach, associated with favorable long-term functional outcomes and a low incidence of hyper-continence. The findings suggest that structured posterior support may play a crucial role in optimizing voiding efficiency and minimizing the need for intermittent catheterization. Future research should focus on comparative studies to further evaluate the benefits of this approach and refine surgical techniques for improved functional recovery.

## Figures and Tables

**Table 1:** Baseline demographics, characteristics, and preoperative features of 32 patients treated with non-organ-sparing RARC + FloRIN reconfiguration.

**Age, median (IQR)**		72 (67–75)
BMI, median (IQR)		24 (22–27)
Smoking status	Never smokers, n (%)	7 (21.8)
	Former smokers, n (%)	20 (62.6)
	Current smokers, n (%)	5 (15.6)
Neo-adjuvant chemotherapy, n (%)		23 (71.9)
CCI age-adjusted, median (IQR)		3 (3–4)
Previous abdominal surgery, n (%)		18 (56.3)
Hydronephrosis observed at CT scan, n (%)		0 (0)
cT ≤ 2, n (%)		28 (87.5)
cT ≥ 3, n (%)		4 (12.5)
cN0, n (%)		29 (90.6)
cN+, n (%)		3 (9.4)
Preoperative urinary incontinence, n (%)		3 (9.4)
Preoperative Pelvic Organ Prolapse ≥ III, n (%)		2 (6.3)

**Table 2:** Intraoperative and perioperative features of 32 patients treated with non-organ-sparing RARC + FloRIN reconfiguration.

**Intraoperative complications, n (%)**	0 (0)
Intraoperative blood transfusions, n (%)	0 (0)
Conversion to open surgery, n (%)	0 (0)
Console time (minutes), median (IQR)	331 (308–347)
Estimated Blood Loss (mL), median (IQR)	330 (260–390)
Time to canalization (days), median (IQR)	5 (4–7)
Stentless procedure, n (%)	5 (15.6)
Hemoglobin level decrease (g/dL), median (IQR)	2.7 (1.8–3.5)
Δ creatinine between baseline—3rd POD, median (IQR)	0.5 (0.2–1.2)
Δ creatinine between discharge and 3rd month assessment, median (IQR)	0.12 (0.03–0.41)
Δ creatinine between discharge and 6th month assessment, median (IQR)	0.11 (0.04–0.43)
Early major (Clavien Dindo ≥ 3) complications (≤30 days), n (%)	3 (9.4)
*Nephrostomy placement*	*2 (6.5)*
*Ileo-ileal anastomosis revision*	*1 (3.1)*
Delayed major (Clavien Dindo ≥ 3) complications (>30 days), n (%)	4 (12.5)
*Nephrostomy placement*	*3 (9.4)*
*Pneumatic dilatation of the uretero-ileal anastomosis*	*1 (3.1)*
Presence of hydronephrosis at 3rd month follow-up assessment, n (%)	2 (6.3)
Readmission rate 30-day, n (%)	2 (6.3)
Median follow-up time, months (IQR)	36 (30–42)

**Table 3:** Functional and urodynamic features of 32 patients treated with non-organ-sparing RARC + FloRIN reconfiguration.

**Hyper-continence status, n (%)**		3 (9.4)
Median self-catheterization number, median (IQR)		3 (2–5)
Urodynamic evaluation	First desire (mL), median (IQR)	190 (170–240)
	Qmax mL/s, median (IQR)	17 (14–25)
	Qave mL/s, median (IQR)	11 (7–18)
	Compliance mL/cmH_2_O, median (IQR)	22 (18–25)
	Abdominal leak points, n (%)	9 (28.1)
	Abdominal leak points minimum volume (mL), median (IQR)	175 (145–230)
	Abdominal leak points minimum pressure (cmH_2_O), median (IQR)	47 (38–55)
	Phasic neobladder contractions, n (%)	8 (25)
	Maximal urethral closure pressure (cmH_2_O), median (IQR)	44 (32–58)
	Post Voidal Residual cc, median (IQR)	40 (30–55)

**Table 4:** Univariate and multivariate analyses assessing clinical predictors of postoperative hyper-continence.

Variable		Univariate Analysis				Multivariate Analysis			
		OR	*p*	95% CI		OR	*p*	95% CI	
				Lower Bound	Upper Bound			Lower Bound	Upper Bound
BMI (kg/m^2^)	>25	1.46	0.02	1.05	1.57	1.24	0.03	1.12	1.39
	≤25	-	-	-	-	-	-	-	-
Age	>70 years	1.35	0.03	1.10	1.66	1.18	0.04	1.09	1.73
	50–69 years	1.21	0.04	1.15	1.89	1.10	0.15	0.97	1.62
	<50 years	-	-	-	-	-	-	-	-
ASA score	>3	1.12	0.04	1.15	1.92	1.02	0.23	0.98	1.11
	≤3	-	-	-	-	-	-	-	-

## Data Availability

The data presented in this study are available on request from the corresponding author due to privacy and confidentiality restrictions.
